# Continuity Is Essential: The Experiences of Co‐Design Participants During the Implementation of a New Health Service

**DOI:** 10.1111/hex.70436

**Published:** 2025-09-19

**Authors:** Deirdre McGowan, Claire Morley, Emily Hansen, Kelly Shaw, Tania Winzenberg

**Affiliations:** ^1^ Menzies Institute for Medical Research University of Tasmania Hobart Australia; ^2^ School of Nursing, College of Health and Medicine University of Tasmania Hobart Australia; ^3^ School of Social Sciences University of Tasmania Hobart Australia; ^4^ KP Health Hobart Australia; ^5^ Primary Health Tasmania Hobart Australia

**Keywords:** co‐design, health services, implementation, primary healthcare, qualitative

## Abstract

**Objective:**

To explore the experiences of participants in the co‐design of a community‐based health service for people with frequent hospital admissions with regard to the design process and its outcomes.

**Study Setting and Design:**

Qualitative semi‐structured interview study with co‐design participants who participated in co‐design meetings during service implementation. Co‐design members were healthcare workers, healthcare consumer representatives, and staff in health services management or leadership roles. Co‐design activities consisted of online meetings, out‐of‐session communication and two facilitated face‐to‐face workshops, which included case studies and small group activities. Health consumer representatives were also contacted out of session to enable them to contribute anything that they thought was important. An interview guide was used, which covered participants' experiences and perceptions of aspects and outcomes of the co‐design process during service implementation. Interviews were online, audio‐visually recorded, transcribed verbatim, and checked for accuracy. Two investigators independently and iteratively analysed data using components of grounded theory.

**Participants:**

Inclusion criteria were being involved in the co‐design process during service design and service implementation, or being involved in the co‐design process after service design, and attending two or more meetings. After applying these criteria, eight people were approached, of whom five agreed to participate.

**Results:**

Participants were interviewed between December 2023 and February 2024. Two themes were identified: (i) fluctuations in co‐design activity impacted engagement and (ii) continuity of co‐design processes across service design and service implementation. These themes are integrated into the final theoretical proposition: continuity in co‐design is essential.

**Conclusion:**

The continuity of people and the continuity of the application of co‐design processes are essential to ensure there is a link between design, implementation and evaluation; a ‘break in the chain’ can impact service implementation. People using co‐design could consider ways to maintain engagement during fluctuations in co‐design activity. Strategies to minimise co‐design team membership turnover may be needed, or, where turnover is unavoidable, ways to effectively onboard new co‐design participants. The continuation of co‐design processes from service design to service implementation may help ensure that there is a link between the co‐design phases. Lack of continuity of co‐design processes may lead to a lack of consistent application of co‐design principles.

**Patient or Public Contribution:**

This was an exploration of the experiences of co‐design participants involved in the design of a new service during the implementation of the service. The co‐design team included representatives from a university, primary healthcare, acute care services and health consumers. This study was informed by previous qualitative interviews with co‐design participants, including two health consumers. These interviews were undertaken during the design phase of the co‐design, and their findings were presented to co‐design participants (written plain language and an oral presentation), and they gave feedback on these. Two co‐design participants were regularly involved in discussions about the research methods, in particular, recruitment and timing of the research. A written summary of the findings from interviews during service implementation was shared with participants, who had an opportunity to provide feedback. One co‐design participant contributed to the ‘Discussion’ section of the manuscript, in particular the implications arising from this study.

## 1 Background

High healthcare service utilisation is where a small portion of the population disproportionately use high amounts of healthcare services and/or expenditure [1, 2, 3]. It is a global phenomenon and is often a marker of people's medical complexity, functional limitations and unmet psychosocial needs [1, 4, 5, 6]. One important manifestation of high healthcare service utilisation is frequent hospital admissions. Scrutiny of people who are frequently hospitalised and interventions targeting this population have proliferated in efforts to reduce avoidable or modifiable admissions [3]. However, such interventions have had limited effectiveness in terms of reducing healthcare utilisation and expenditure [3, 7], possibly due to limited involvement of people with lived experience [8, 9]. Consequently, there is increasing recognition that people with lived experience of frequent hospital admissions should be involved in the design of services and interventions that are specifically for them [5, 8, 10].

Co‐design is one possible way to give voice to people with lived experience to ensure services meet the needs of the people for whom they are designed [11, 12]. It is the process of bringing end‐users (consumers, community members, healthcare workers, and policymakers) together to (re)design health services or quality improvements [12, 13] and has the potential to create person‐centred, high‐value healthcare [9, 13, 14]. There are many approaches to co‐design, which may be attributable to needing flexibility to tailor co‐design to the circumstances of individual programmes [15]. However, this flexibility has led to a proliferation of co‐design definitions, principles and processes [15, 16], leading to inconsistent reporting [14, 15, 17] and a weak evidence base [14, 15, 17, 18, 19, 20]. Moreover, there has been ambiguity and misuse of co‐design terminology in projects that do not involve consumers or community members and thus are not co‐designed or co‐produced, but rather are consultative design [16].

Co‐design involves distinct, but not mutually exclusive, phases of design, implementation and evaluation [12]. A recent systematic review of strategies to involve people in co‐design found that formal evaluations of co‐design engagement experiences were scarce [14]. Moreover, the stage of co‐design being reported can be unclear [14], thus whether co‐design processes differ between co‐design phases is poorly reported. Understanding co‐design during service implementation is important because this is when the design meets the ‘real world’ [21, 22], yet few studies have explored co‐design participants' experiences during service implementation or made distinctions between co‐design phases to examine whether co‐design processes change over time [23, 24].

We previously reported on co‐design participants' experiences during the design phase of a new service for people with frequent hospital admissions [10]. The aim of this study was to further explore participants' experiences, perceptions and attitudes to the design process and co‐design outcomes during service implementation.

## 2 Materials and Methods

An approach drawing on aspects of grounded theory [25] and principles of qualitative rigour [26] informed the design of this semi‐structured interview study. Grounded theory was chosen because it places participants in the centre of the research question, findings are rooted in the data, and it provides a rigorous, iterative framework for data collection, analysis and interpretation [25]. The components of grounded theory used were concurrent data collection and analysis, constant comparison of new and existing data using codes and themes to iteratively generate concepts (constant comparative analysis), documenting and reflecting on the analysis process (memoing), three levels of iterative coding (initial, intermediate and theoretical coding), using semi‐structured interviews to follow data leads provided by participants (theoretical sampling), and identifying what is meaningful for theory development while also not imposing fixed ideas or researchers past experiences on data analysis (theoretical sensitivity) [25]. The first two levels of coding—initial and intermediate—can be likened to thematic analysis, whereby the data is fractured (initial coding) and then transformed into themes (intermediate coding) [25]. The final stage of coding (theoretical coding) provides the researchers with an opportunity to integrate the codes developed in the previous stages and to pull together the analysis to identify a theoretical thread that explains what is happening in the data. This process results in a final theoretical proposition, which is grounded in the data collected [25].

Reporting of qualitative procedures were guided by the COnsolidated criteria for REporting Qualitative research (COREQ) [27] (Supplement 1). The all‐female research team comprised two registered nurses (D.M. and C.M.), one sociologist (E.H.) and two general practitioners (GPs) (K.S. and T.W.). The study received ethical approval from the Human Research Ethics Committee of the University of Tasmania, Australia (H0026675).

### 2.1 Setting and Participants

In Australia, the Federal Government funds 31 independent, not‐for‐profit organisations, called Primary Health Networks, to manage, commission and tailor primary healthcare to regions across Australia [28]. Primary Health Tasmania is one such Primary Health Network, which led the co‐design of a new, community‐based health service for people with frequent hospital admissions in Tasmania, Australia. The co‐designed service was a nurse‐led, multidisciplinary, community‐based service, which comprised a nurse manager, social worker, allied health assistant, GP and administration assistant.

Co‐design commenced in early 2021 during the Covid‐19 pandemic, with a team of 26 people across three governance structures: a Steering Group, Advisory Group and Working Group. Co‐design team membership comprised of healthcare workers, healthcare consumer representatives and staff in health services management or leadership roles, coming from organisations including Primary Health Tasmania, the Tasmanian Department of Health, the Tasmanian Health Service, the University of Tasmania, private general practices and Health Consumers Tasmania. The Covid‐19 pandemic affected the type and timing of co‐design activities, which consisted of online meetings of Steering, Advisory or Working Groups, out‐of‐session communication (email and one‐on‐one meetings), and two face‐to‐face workshops, which included case studies and small group activities (March and April 2021). In August 2021, the Advisory Group and Working Group merged to form a combined Advisory Group. In late 2022, design transitioned into service implementation, and the team decreased to 20 people comprising healthcare workers, healthcare consumer representatives and staff in health services management or leadership roles. Participants varied in the duration of time they participated in the co‐design. The co‐design process during implementation consisted of online meetings only. At the time interviews commenced, the service had been operational for 17 months.

Face‐to‐face workshops were facilitated by a person experienced in facilitating co‐design groups and workshops with diverse participants, including consumers, health providers and policymakers, to ensure potential power imbalances were addressed, equal participation of all participants, including consumers, and building of a cohesive co‐design team to work together on service design. The face‐to‐face workshops included presenting case studies as examples of potential clients of the service to stimulate discussion by the group and a plain language presentation of relevant research evidence with opportunities for questions to support the design process. Similar approaches were used during online meetings. Health consumer representatives were also contacted individually outside of meetings (via telephone), so they were not constrained by the group environment, checking in that they were happy with the group process and providing space for them to talk about and contribute anything that they thought was important.

Potential participants were invited to participate if they met one of the following inclusion criteria: they had participated in the design phase interviews and were still involved in the co‐design process; they declined to participate in the design phase interviews but were still involved in the co‐design process; or if they began participating in the co‐design process after the design phase and had attended two or more meetings. One GP who was a co‐design participant and an investigator in this study was excluded. After applying the inclusion criteria, seven co‐design participants who were present from the beginning of co‐design and one participant who joined partway through were approached. Participants were sent an email on behalf of the research team by a Primary Health Tasmania delegate. Participants who did not respond within 2 weeks received a follow‐up email and/or phone call from a member of the research team.

### 2.2 Data Collection and Analysis

A semi‐structured interview guide (Supplement 2) was developed to explore participants' experiences and perceptions of the co‐design process and its outcomes over the preceding 12 months during service implementation. Participants took part in one semi‐structured interview. Data collection and analysis occurred concurrently between December 2023 and February 2024, and data saturation was reached. As preferred by participants, interviews were undertaken online at their workplace. No one else was present during the interviews. Interviews were audio‐visually recorded and transcribed verbatim via inbuilt transcription software, and transcriptions were checked for accuracy. Participants received a copy of their transcript and were able to make changes if they wished.

Two researchers (D.M. and C.M.) iteratively analysed data using NVivo Version 1.7.1 (1534), initially independently, and then met to discuss each other's perspective, any data leads that arose during data analysis that needed following up, and collaboratively decided on codes, themes and analytical concepts that explained what was happening in the data. The whole research team met at regular intervals to refine the analysis. An audit trail was maintained after each interview and when reflecting on the data to document evolving analysis and themes. Constant comparative analysis was used to develop a theoretical proposition. Participants received a plain word summary of findings and were offered the opportunity to provide feedback if they wished.

## 3 Findings

Of eight potential participants approached, five agreed to participate, all of whom had been interviewed in the design phase. The average interview length was 26 min. Due to the small sample size, characteristics of the participants are not provided to maintain their confidentiality, but the participants were drawn from the study cohort described elsewhere [10].

Our analysis identified two themes: (i) fluctuations in co‐design activity impacted engagement and (ii) continuity of co‐design processes across service design and service implementation. Themes, sub‐themes and codes are presented in Table 1. Themes and sub‐themes are described in more detail below, and example quotes are provided in Table 2. The two themes integrated into the final theoretical proposition: continuity in co‐design is essential. We identified that continuity of people and continuity of application of co‐design processes are both essential to ensure there is a link between design, implementation and evaluation; a ‘break in the chain’ can impact service implementation.

**Table 1:** Themes, sub‐themes and codes of data from participant interviews.

Theme	Sub‐theme	Code
Fluctuations in co‐design activity impacted engagement	n/a	Peaks and lulls in co‐design activity
		‘Co‐design work versus co‐design process’
		Feeling frustrated by and disconnected from the co‐design process
Continuity of co‐design processes across service design and service implementation	Changing the co‐design team membership affected continuity and commitment	Co‐design team membership turnover
		Relationships can be enabled or threatened
	Believe in co‐design	Believe in co‐design
		Case studies were helpful to shape understanding and improve the design
	Differences between what was imagined and what eventuated	No one really knew how it would go
		Uncertain about some design elements and long‐term sustainability
		Differences in client recruitment, numbers, care needs and staff needed to provide care
		Important components to get right

**Table 2:** Themes, sub‐themes and example quotes from participant interviews.

Sub‐theme	Code	Example quotes
Fluctuations in co‐design activity impacted engagement		
n/a	Peaks and lulls in co‐design activity	So, when I think about the last 12 months in terms of the co‐design process, it doesn't feel like we have [co‐designed]…. But given where we're at in the work [implementation], is that OK? I think that's the question for me. [participant] …to more clearly be able to describe you know, the peaks and lulls in when we're, I guess, more formally in a co‐design phase or needing some co‐design investment … being clearer that there may actually be less need for co‐design during that [implementation] phase, so that we don't have an advisory group potentially sitting there going ‘where have you all gone, you know, what are we co‐designing?’ [participant]* We're in it [co‐design] for the long haul and … we have a genuine commitment to it. But how that engagement, what it looks like and the intensity of it varies over that time. [participant]
	‘Continuous co‐design process versus continuous co‐design actual work’	…like a continuous co‐design process as opposed to continuous co‐design actual work, if that makes sense … what are the things we can continue to do through a co‐design process that would mean we are keeping our eyes open to other perspectives … how do we make sure that we're not missing potential co‐design opportunities? [participant] Once a service is into the system, once you've gone through a process of saying this is what we're going to purchase, this is what we need you to deliver, your ability to actually make major changes is much more limited. So, to me, the essence of co‐design lies in the process that sits before you actually put a service on the ground, because changing a service once it's there, is only a fractional gain. You can change elements, you can change the balance of particular elements within a service model, but the service model itself is what's essentially set up beforehand. [participant]
	Feeling frustrated by and disconnected from the co‐design process	I feel like I've been disconnected. [participant] …the process theoretically is good, but how it's actually happened and what's actually happened is opaque. [participant]
Continuity of co‐design processes across service design and service implementation		
Changing the co‐design team membership affected continuity and commitment	Co‐design team membership turnover	I'd say in many respects, co‐design has given way to this need to actually try and retrain and re‐link new people to an existing service model that they don't feel an ownership of, or they don't feel as though they've actually had the opportunity to help shape themselves. And so, you end up with varying levels of commitment and varying levels of understanding and awareness about what's trying to happen…. I have found it incredibly frustrating to continuously be dealing with the same issues around commitment, awareness and understanding across the board in this particular program, it's been very, very difficult. [participant] I think one of the learnings for me is how you maintain that sense of understanding of the vision and what we're trying to achieve and the history and steps that have been taken to date in a process so that when you do get that change in [team membership], there's ways you can try and bring people up to speed quickly and get some of that understanding to then be able to help make informed decisions. [participant] …that for me has been a bit frustrating in that you put a lot of work and effort in, and you think that people are on board and then you hit a speed hump and there's not shared understanding that we need to work together to get over it. It's more, there's a more potentially passive view of the importance of trying to resolve some of the issues. [participant]
	Relationships can be enabled or threatened	You don't ever want to be in a situation where you're wagging your finger [participant] I think that focus on trying to develop a really strong collaborative approach has helped us to be able to work through [issues]…. We've developed that relationship that we can have some of those frank and open conversations. [participant]
Believing in co‐design	Believing in co‐design	It's interesting to hear other people's perspectives and even their perceptions. [participant] We've certainly had some bumps in the program and some learnings in the implementation, but I think that focus on trying to develop a really strong collaborative approach has helped us to be able to work through that and I hope for [all parties] to be comfortable in raising where they're having challenges or issues or where we're seeing issues that we've got. [participant] I think the patients have really had [a] good service. I think they've had some fantastic support from a really committed group of people. [participant]
	Case studies were helpful to shape understanding and improve the design	It's very easy in any sort of service to just reduce the information to the basic data. You know, saying this many clients with this amount of time and it can look like it's not particularly efficient. But when you do hear those individual client stories, it does I think help partner awareness of how long it takes to effect change, how much hard work it is and how time‐consuming it is, and the complexity of issues that a lot of these patients are affected by. [participant] So [the case studies have] been a really good feature and we've used that as a way of externally describing how the service works to others and reinforcing its value. [participant]
Differences between what was imagined and what eventuated	No one really knew how it would go	Things don't always go according to the vision and what you think you're going to be able to achieve … essentially, we're designing something new, different, and we might have a sense of how we would like it to work. But then practicalities like just recruitment of workforce and things like that become challenging and doesn't matter how much you wish for it, you can't magic up workforce. [participant] I think being a new service, no one really knew how it was supposed to run. What it was supposed to do, and so that always means that you've got a long lag time … it's not going to be as productive initially. [participant]
	Uncertain about some design elements and long‐term sustainability	We've been dealing with a level of [client] complexity that means that ordinarily, providing services for this type of client group in a general practice environment would be all but impossible. They don't have the nursing hours, they don't have the social work support or other supports available through an allied health team…. So, my view would be this service has no chance of being sustainable. [participant] The project is not sustainable because there isn't a funding source for it … the state [hospital] doesn't receive payment for patients unless they're in a bed. This service takes people out of beds and says here you can use that bed for somebody else. They'll get a payment for that patient and their service, but they don't get any resourcing to service patients that they take out of [a] bed. [participant]
	Differences in client recruitment, numbers, care needs and staff needed to provide care	If you look, for example, at the number of patients that are eligible, you think hundreds, this is great. But actually, when you start to go through that, actually a significant proportion of these are … not eligible for the service, so you end up with a lot smaller number of clients than we were originally anticipating. So, then you've got to try and actively recruit or reconsider changing your criteria for service selection. [participant] We always knew [social work and nursing] was going to be a big feature, but we didn't know that it was going to be quite at the level that it was with the type of client group that we're dealing with. [participant] [I] probably thought there would be some social benefits, but they're probably a lot stronger than I thought. [participant]
	Important components to get right during co‐design	I don't know that there was much consumer involvement from consumers, carers, families involved in the process. And maybe there should have been two levels. One was about the broad concept of the service, and then maybe there should have been a second one which was about what consumers, carers, families and some of those key staff, bringing them together to [identify] what exactly do you want and need, and how are we going to deliver that. [participant] I think that design pre the commencement of the service was probably very theoretical and looking a bit at the data. It probably didn't drill down to service delivery level, perhaps quite enough. [participant] There wasn't enough focus given … to where this service fitted within [the] clinical hierarchy to ensure that good clinical governance was being applied, and consequently, I think this team was left out on the periphery rather than bought in as part of the mainstream service offering. [participant]

* Quotes from participants. Participant identifiers have not been used to maintain confidentiality and anonymity.

## 4 Fluctuations in Co‐Design Activity Impacted Engagement

The first theme is that fluctuations in co‐design activity impacted engagement. Codes are described below with key illustrative quotes.

Most participants described changes in the intensity of their involvement as the service moved into the implementation phase. Participants described their involvement as ‘big upfront [design phase], smaller in the middle [implementation phase], and big at the back end [evaluation phase]’ and that there were ‘less equal and reciprocal relationships and more checking‐in [and] updating’ during service implementation (Table 2). Some participants felt that responsibility shifted away from co‐design participants towards funders and those within the health system or operationalising the service. The fluctuations in co‐design activity made some participants feel the co‐design process was opaque and that they were disconnected from it (Table 2). As a result, they described feeling that they were unable to advocate for the service with industry partners and community members (Table 2).

Participants reflected on the co‐design activity over time as the service was operationalised.

‘When I think about the last 12 months in terms of co‐design process, it doesn't feel like we have [co‐designed] … we haven't been actively doing that type of work. But given where we're at in the work, is that OK? I think that's the question for me. Or do we need to be looking at how we're doing [co‐design]—you see, this is where I get stuck, like a continuous co‐design process as opposed to continuous co‐design actual work, if that makes sense. We're not in a position at the moment where we've got pieces of work to come back and go right, we really need to flesh this out…. But what are the things we can continue to do through a co‐design process that would mean we are keeping our eyes open to other perspectives and other thoughts of how we can be implementing’.

One participant thought that during lulls in co‐design activity, continuation of co‐design processes could be used to allow co‐design participants to more deeply explore some service design elements, such as ‘deep dives’, which may lead to other co‐design opportunities and help to keep the co‐design team engaged and informed (Table 2).

## 5 Continuity of Co‐Design Processes Across Service Design and Service Implementation

The second theme comprises three sub‐themes: (i) changing co‐design team membership affected continuity and commitment, (ii) believing in co‐design and (iii) differences between what was imagined and what eventuated. Sub‐themes are described below with key illustrative quotes.

### 5.1 Changing Co‐Design Team Membership Affected Continuity and Commitment

Most participants described a high rate of co‐design team membership turnover, which they felt negatively affected their experience. Some participants felt that new team members did not have ownership of the co‐designed service, which resulted in the team being ‘champion‐less’ within some project partners (Table 2).

‘Definitely different expectations, different understandings about roles and responsibilities. Different understanding about the objective and the intention behind the service. Different understanding about why it's actually running from within the hospital sector and not running in a community environment. Right across the board, there's differences in awareness, understanding and this cycling through [of] people has not helped to engender any sense of, you know, embedded responsibility, obligation or clarity in role and responsibility’*.*

Some participants felt that once shared commitment and understanding was lost, it was very difficult to regain, which some felt could threaten relationships with other co‐design team members (Table 2). One participant thought that there could be better ways to rapidly onboard new team members to maintain continuity.

### 5.2 Believing in Co‐Design

Participants believed in co‐design and spoke about opportunities and positive outcomes as a result of co‐design processes that continued during service implementation. These included hearing other people's perspectives, the opportunity to develop strong collaborative relationships and inform future co‐design work and models of care through evaluation (Table 2). Participants described anonymised client case studies presented by a staff member of the service as helpful, and one participant thought it provided indirect consumer involvement. Participants described how case studies had given them an understanding of how the service was operating and the needs and outcomes of clients, which helped them to identify design successes and areas for improvement (Table 2). Others found the case studies helpful in describing the service externally and guiding them in related areas of work. Some participants felt that there was ‘some really good evidence to say this is how we would do it’ based on ‘evaluation in motion’ of the service that had been occurring during co‐design (Table 2).

### 5.3 Differences Between What Was Imagined and What Eventuated

Most participants, but not all, believed the service was the right model of care. They felt the service was flexible, person‐centred, and impactful for clients. Many were uncertain about some design elements and whether the service was sustainable long‐term. Participants described uncertainty related to insecure funding sources and felt that the new service was restricted by the system in which it was operating (Table 2). Some participants were uncertain about design elements, such as how the service GP role fit within clients' existing primary care teams and how intensive multidisciplinary care was sustainable long‐term in general practice (Table 2).

‘If this [client] struck up a therapeutic relationship with this [staff member] that's not general practice and it's not their GP, how's this all going to translate into reality down the track? The impression gained [from the case study provided by a staff member] was that the [client] had complexities and had sort of not been engaging … but who in reality down the track's going to pay that sort of time for that sort of person to develop that sort of relationship with people … is it sustainable? Is it realistic?…. I wondered about sustainability … the [staff member] involved who [was] reporting back was really happy, and I can understand that, but you've got to think about the longer term, and how is it all going to work’.

Participants described service implementation over time, that things don't always go to plan, and because it was a new service, no one really knew how the service would work (Table 2). Participants described gaps in co‐design processes, such as involving consumers or people with lived experience, but the effects of these gaps were not fully realised until the implementation phase. This resulted in differences between how the service was imagined and what eventuated. Such differences included adaptations to the client recruitment process, the overestimation of potential client numbers, and an underestimation of the intensity of support clients would require and therefore the amount of staff resources required to provide this care (Table 2). Some participants believed that some of the differences between what was imagined and what eventuated were due to limited involvement of consumers or people with lived experience of frequent hospital admissions (Table 2).

‘I don't know that there was that much consumer involvement from consumers, carers, families involved in the [co‐design] process and maybe there should have been two levels. One was about the broad concept of the service, and then maybe there should have been a second one which was about consumer, carers, families and some of those key staff bringing them together to [identify] “what exactly do you want and need, and how are we going to deliver that?”’

Others spoke about delays in implementation. Some participants thought this was due to a lack of consideration of what was needed for implementation during the design phase or because pre‐implementation work that had been done became out‐of‐date due to delays in implementation (Table 2). Participants described how gaps in implementation processes impacted service implementation, such as unclear work processes and key performance indicators. Some participants thought that leadership and managerial support from some industry partners could have been improved during the co‐design process. Others spoke about other implementation issues, such as unclear clinical governance for clinical staff, and lack of incorporation of the new service into clinical hierarchy and mainstream processes (Table 2).

## 6 Discussion

To our knowledge, this is the first study to explicitly explore the experiences of co‐design participants during implementation of a co‐designed service. Two themes were identified: (i) fluctuations in co‐design activity impacted engagement and (ii) continuity of co‐design processes across service design and service implementation. These are integrated into the final theoretical proposition: continuity in co‐design is essential. These findings have implications for those undertaking co‐design. First, where the need for participant involvement in co‐design fluctuates, consideration could be given to strategies that maintain engagement and involvement, such as ‘deep dives’ into design elements. Second, consideration could be given to strategies that reduce team membership turnover, or if turnover is unavoidable, ways to effectively and efficiently onboard new co‐design participants could be explored. Third, the continuation of co‐design processes from service design to service implementation may help to ensure that there is a link between co‐design phases. Fourth, the lack of continuity of co‐design processes potentially leads to a lack of consistent application of co‐design principles. Applying these findings could help with successful service implementation and help to ensure that co‐designed services are sustainable. Overall, continuity of people and continuity of application of co‐design processes are both essential to ensure there is a link between design, implementation and evaluation; a ‘break in the chain’ can impact service implementation.

Participants described a lack of continuity of people in terms of fluctuations in the intensity of design activity. They felt that fluctuations in the level of co‐design activity affected engagement, leading to some participants feeling disconnected from the co‐design process. Gaps in communication of co‐design processes and outcomes leading to participants feeling frustrated have been highlighted in other studies [20, 29, 30, 31, 32]. Our findings provide further support that co‐design participants should be made aware of the fluctuations in activity that may occur and that those using this approach could take steps to keep co‐design participants engaged and involved. ‘Deep dives’ into design elements was suggested by one participant as a way to maintain co‐design activity and processes during lulls in activity. Co‐design principles and connection to the people at the heart of the issue at hand through a stated purpose and the involvement of people with lived experience have also been suggested as possible ways to maintain continuity during the implementation of co‐designed services [12, 20, 24, 33]. However, the best approach to this is not clear, and more research is required to determine how co‐design teams can be kept engaged and committed during fluctuations in activity [24].

Participants expressed frustration at co‐design team membership turnover that occurred for members across a range of the participating organisations and challenges in onboarding new co‐design team members. They felt co‐design team membership turnover affected shared ownership, momentum and engagement. Team membership turnover and its effects have been commonly reported in other co‐design studies [21, 24, 33, 34, 35, 36, 37]. Lack of shared purpose and ownership can result in healthcare workers perceiving co‐design processes and decisions through a competitive mindset, whereby there is competition for resources, power and legitimacy, rather than opportunities for collaboration, innovation and better outcomes for the people at the heart of the issue at hand [24, 38, 39]. Our findings suggest that co‐design teams may be unsure of how to effectively onboard new team members. Consideration could be given to strategies that reduce team membership turnover or, if turnover is unavoidable, strategies that effectively onboard new co‐design participants. Such strategies include orientation, support and training, which have been suggested as helpful in onboarding new team members to existing teams in other industries [40, 41]. However, there is little specific guidance for co‐design teams in health services, and this requires further investigation.

Participants valued co‐design processes that continued during service implementation, such as hearing other perspectives and listening to case studies and collaborative relationships. However, they also felt that there were some gaps in some co‐design processes. Such gaps included perceptions of incomplete implementation processes, which led to implementation delays, or limited involvement of people with lived experience. This co‐design had additional challenges stemming from the Covid‐19 pandemic, which may have affected co‐design processes. Similar findings were reported in a study of healthcare workers implementing a co‐designed youth mental health service, where service implementation took longer and consumers involved in co‐design were not representative of people who were using the service [22]. Our findings suggest that the application of all co‐design processes contributes to service implementation. However, previous co‐design and experience‐based co‐design systematic reviews have found that application and fidelity of co‐design processes vary [15, 16, 42, 43].

Participants also described continuity of co‐design processes, or lack thereof, that impacted consistent application of co‐design principles, such as *equal partnership, openness, respect, empathy and design together* [12]. In this co‐design, participants described positive outcomes arising from the continuation of co‐design principles and processes, such as having ‘a really strong collaborative approach has helped us to be able to work through [challenges]’ and by doing service ‘evaluation in motion’ has provided ‘really good evidence’. However, participants also felt that there were gaps in the application of co‐design principles and processes, such as not being sure that there was ‘much consumer involvement from consumers, carers, [and] families’ or that ‘there's not shared understanding that we need to work together’. These findings are similar to a recent review of 60 co‐design definitions, which recommend that co‐design processes and their outcomes should match co‐design principles [16]. Our findings suggest that the lack of continuity of co‐design processes potentially leads to the lack of consistent application of co‐design principles. They also provide further support that co‐design principles and processes are not linear, but rather cyclic, where end‐users design, implement and evaluate together, which reinforces that co‐design continues from service design into service implementation [12, 20, 22, 44].

We found that continuity of people and continuity of application of co‐design processes are both essential in co‐design. These findings are in contrast to previous co‐design research, which has described continuity from one perspective only: that of a constant state of team membership [24, 33, 36, 37]. Our findings suggest that continuity in co‐design runs deeper than this. The continuity of people and co‐design processes is essential to ensure there is a link between design, implementation and evaluation; a ‘break in the chain’ can impact service implementation. Explicitly incorporating implementation and evaluation methodologies in co‐design, such as action learning [45, 46] or developmental evaluation [47], may promote links between service design, implementation and evaluation, and go some way to create co‐designed services that are responsive, feasible and sustainable [11, 20, 22, 24, 31, 48].

There are some limitations to our study. We had a small sample size; however, we were able to reach data saturation, so if further interviews were possible, they may not have added greatly to the findings. The exception to this is that we were unable to recruit healthcare consumer(s) who participated in the co‐design, and as such, our study lacks their insights. This study explored the co‐design process of a new service, which is different from re‐design or quality improvement initiatives of existing services, as the client base and service outputs (such as key performance indicators and workflows) are already known. Thus, some findings may not apply to co‐design in these situations. Nonetheless, our study contributes to the limited knowledge regarding the experiences of co‐design participants during service implementation.

## 7 Conclusion

This study identified two important themes from the experiences of co‐design participants during service implementation, namely: (i) fluctuations in co‐design activity impacted engagement and (ii) continuity of co‐design processes across service design and service implementation. These findings have implications for those undertaking this design methodology. Co‐design involvement may fluctuate over time; consideration could be given to strategies that maintain engagement and involvement. Consideration could be given to strategies that reduce team membership turnover, or, where turnover is unavoidable, ways to onboard new co‐design participants. The continuation of co‐design processes from service design to service implementation may help ensure that there is a link between the co‐design phases. Lack of continuity of co‐design processes potentially leads to a lack of consistent application of co‐design principles. Overall, the continuity of people and the continuity of application of co‐design processes are essential to ensure there is a link between design, implementation and evaluation; a ‘break in the chain’ can impact service implementation.

## Author Contributions

**Deirdre McGowan:** conceptualisation, data curation, formal analysis, investigation, methodology, project administration, visualisation, writing – original draft preparation. **Claire Morley:** conceptualisation, formal analysis, investigation, supervision, writing – review and editing. **Emily Hansen:** conceptualisation, formal analysis, methodology, supervision, writing – review and editing. **Kelly Shaw:** conceptualisation, formal analysis, supervision, writing – review and editing. **Tania Winzenberg:** conceptualisation (lead), funding acquisition, methodology, formal analysis, project administration, supervision, writing – review and editing.

## Ethics Statement

The study received ethical approval from the Human Research Ethics Committee of the University of Tasmania, Australia (H0026675).

## Consent

Participants provided verbal or written consent before taking part in this study.

## Conflicts of Interest

Tania Winzenberg—2020—$885 for preparation of educational material related to osteoporosis for AMGEN. The other authors declare no conflicts of interest.

## Data Availability

The data that support the findings of this study are available upon reasonable request from the corresponding author. The data are not publicly available due to privacy or ethical restrictions.

## References

[1] N. D. Berkman , E. Chang , J. Seibert , et al., Management of High‐Need, High‐Cost Patients: A “Best Fit” Framework Synthesis, Realist Review, and Systematic Review Comparative Effectiveness Review No. 246. AHRQ Publication No. 21(22)‐EHC028 (Agency for Healthcare Research and Quality, 2021). 34780127

[2] E. M. Mitchell . “Concentration of Healthcare Expenditures and Selected Characteristics of High Spenders, US Civilian Noninstitutionalized Population, 2019,” in *Statistical Brief (Medical Expenditure Panel Survey (US)*. 2022. Statistical Brief #540.

[3] A. Goodwin , B. L. Henschen , L. C. O'Dwyer , N. Nichols , and K. J. O'Leary , “Interventions for Frequently Hospitalized Patients and Their Effect on Outcomes: A Systematic Review,” Journal of Hospital Medicine 13, no. 12 (2018): 853–859. 30379144 10.12788/jhm.3090

[4] P. Long , M. Abrams , A. Milstein , et al., Effective Care for High‐Need Patients: Opportunities for Improving Outcomes, Value, and Health (National Academy of Medicine, 2017). 37748007

[5] M. Huang , C. Van Der Borght , M. Leithaus , J. Flamaing , and G. Goderis , “Patients' Perceptions of Frequent Hospital Admissions: A Qualitative Interview Study With Older People Above 65 Years of Age,” BMC Geriatrics 20 (2020): 332. 32894056 10.1186/s12877-020-01748-9 PMC7487888

[6] S. L. Hayes , C. A. Salzberg , D. McCarthy , et al., High‐Need, High‐Cost Patients: Who Are They and How Do They Use Health Care? (The Commonwealth Fund, 2016), https://www.commonwealthfund.org/publications/issue-briefs/2016/aug/high-need-high-cost-patients-who-are-they-and-how-do-they-use. 27571599

[7] C. Hudon , M.‐C. Chouinard , M. Lambert , I. Dufour , and C. Krieg , “Effectiveness of Case Management Interventions for Frequent Users of Healthcare Services: A Scoping Review,” BMJ Open 6, no. 9 (2016): e012353. 10.1136/bmjopen-2016-012353 PMC5051491 27687900

[8] K. J. O'Leary , M. M. Chapman , S. Foster , L. O'Hara , B. L. Henschen , and K. A. Cameron , “Frequently Hospitalized Patients' Perceptions of Factors Contributing to High Hospital Use,” Journal of Hospital Medicine 14, no. 9 (2019): 521–526. 30897060 10.12788/jhm.3175 PMC6715053

[9] L. K. Wiles , D. Kay , J. A. Luker , et al., “Consumer Engagement in Health Care Policy, Research and Services: A Systematic Review and Meta‐Analysis of Methods and Effects,” PLoS One 17, no. 1 (2022): e0261808. 35085276 10.1371/journal.pone.0261808 PMC8794088

[10] D. McGowan , C. Morley , E. Hansen , K. Shaw , and T. Winzenberg , “Experiences of Participants in the Co‐Design of a Community‐Based Health Service for People With High Healthcare Service Use,” BMC Health Services Research 24 (2024): 339. 38486164 10.1186/s12913-024-10788-5 PMC10938828

[11] C. Morley , K. Jose , S. E. Hall , et al., “Evidence‐Informed, Experience‐Based Co‐Design: A Novel Framework Integrating Research Evidence and Lived Experience in Priority‐Setting and Co‐Design of Health Services,” BMJ Open 14, no. 8 (2024): e084620. 10.1136/bmjopen-2024-084620 PMC11404138 39122385

[12] Agency for Clinical Innovation . Patient Experience and Consumer Engagement—A Guide to Build Co‐Design Capability. Chatswood, NSW, Engagement PEaC. 2019.

[13] Australian Commission on Safety and Quality in Health Care . Partnering With Consumers in Organisational Design and Governance: Consumers Are Partners in the Design and Governance of the Organisation. 2024.

[14] Y. Bombard , G. R. Baker , E. Orlando , et al., “Engaging Patients to Improve Quality of Care: A Systematic Review,” Implementation Science 13, no. 98 (2018): 98. 30045735 10.1186/s13012-018-0784-z PMC6060529

[15] T. Greenhalgh , L. Hinton , T. Finlay , et al., “Frameworks for Supporting Patient and Public Involvement in Research: Systematic Review and Co‐Design Pilot,” Health Expectations 22, no. 4 (2019): 785–801. 31012259 10.1111/hex.12888 PMC6737756

[16] D. Masterson , K. Areskoug Josefsson , G. Robert , E. Nylander , and S. Kjellström , “Mapping Definitions of Co‐Production and Co‐Design in Health and Social Care: A Systematic Scoping Review Providing Lessons for the Future,” Health Expectations 25, no. 3 (2022): 902–913. 35322510 10.1111/hex.13470 PMC9122425

[17] N. Lloyd , A. Kenny , and N. Hyett , “Evaluating Health Service Outcomes of Public Involvement in Health Service Design in High‐Income Countries: A Systematic Review,” BMC Health Services Research 21, no. 1 (2021): 364. 33879149 10.1186/s12913-021-06319-1 PMC8056601

[18] D. Clarke , F. Jones , R. Harris , and G. Robert , “What Outcomes Are Associated With Developing and Implementing Co‐Produced Interventions in Acute Healthcare Settings? A Rapid Evidence Synthesis,” BMJ Open 7, no. 7 (2017): e014650. 10.1136/bmjopen-2016-014650 PMC5734495 28701409

[19] H. Smith , L. Budworth , C. Grindey , et al., “Co‐Production Practice and Future Research Priorities in United Kingdom‐Funded Applied Health Research: A Scoping Review,” Health Research Policy and Systems 20, no. 1 (2022): 36. 35366898 10.1186/s12961-022-00838-x PMC8976994

[20] É. Ní Shé and R. Harrison , “Mitigating Unintended Consequences of Co‐Design in Health Care,” Health Expectations 24, no. 5 (2021): 1551–1556. 34339528 10.1111/hex.13308 PMC8483209

[21] J. W. Kirk , P. Nilsen , O. Andersen , et al., “Adaptations and Modifications to a Co‐Designed Intervention and Its Clinical Implementation: A Qualitative Study in Denmark,” BMC Health Services Research 21 (2021): 1108. 34656126 10.1186/s12913-021-07142-4 PMC8520628

[22] M. Kehoe , R. Whitehead , K. De Boer , D. Meyer , L. Hopkins , and M. Nedeljkovic , “Are Codesigned Programmes More Difficult to Implement? A Qualitative Study of Staff Perceptions on the Implementation of a New Youth Mental Health Programme,” Health Expectations 27, no. 1 (2024): e13989. 38367246 10.1111/hex.13989 PMC10874247

[23] M. Farr , “Power Dynamics and Collaborative Mechanisms in Co‐Production and Co‐Design Processes,” Critical Social Policy 38, no. 4 (2018): 623–644.

[24] A. Pirinen , “The Barriers and Enablers of Co‐Design for Services,” International Journal of Design 10, no. 3 (2016): 27–42.

[25] Y. Chun Tie , M. Birks , and K. Francis , “Grounded Theory Research: A Design Framework for Novice Researchers,” SAGE Open Medicine 7 (2019): 1–8. 10.1177/2050312118822927 PMC6318722 30637106

[26] G. A. Tobin and C. M. Begley , “Methodological Rigour Within a Qualitative Framework,” Journal of Advanced Nursing 48, no. 4 (2004): 388–396. 15500533 10.1111/j.1365-2648.2004.03207.x

[27] A. Tong , P. Sainsbury , and J. Craig , “Consolidated Criteria for Reporting Qualitative Research (COREQ): A 32‐Item Checklist for Interviews and Focus Groups,” International Journal for Quality in Health Care 19, no. 6 (2007): 349–357. 17872937 10.1093/intqhc/mzm042

[28] Commonwealth of Australian Department of Health and Aged Care . Primary Health Networks: Commonwealth of Australia. 2022, https://www.health.gov.au/our-work/phn#:~:text=Australia's%2031%20Primary%20Health%20Networks,place%2C%20at%20the%20right%20time.

[29] K. S. Pallesen , L. Rogers , S. Anjara , A. De Brún , and E. McAuliffe , “A Evaluation of Participants' Experiences of Using Co‐Design to Develop a Collective Leadership Educational Intervention for Health‐Care Teams,” Health Expectations 23, no. 2 (2020): 358–367. 31999883 10.1111/hex.13002 PMC7104638

[30] S. Lindblom , M. Flink , M. Elf , A. C. Laska , L. Von Koch , and C. Ytterberg , “The Manifestation of Participation Within a Co‐Design Process Involving Patients, Significant Others and Health‐Care Professionals,” Health Expectations 24, no. 3 (2021): 905–916. 33729653 10.1111/hex.13233 PMC8235880

[31] G. Mulvale , S. Moll , A. Miatello , et al., “Codesigning Health and Other Public Services With Vulnerable and Disadvantaged Populations: Insights From an International Collaboration,” Health Expectations 22, no. 3 (2019): 284–297. 30604580 10.1111/hex.12864 PMC6543156

[32] É. Ní Shé , J. Cassidy , C. Davies , et al., “Minding the Gap: Identifying Values to Enable Public and Patient Involvement at the Pre‐Commencement Stage of Research Projects,” Research Involvement and Engagement 6 (2020): 46. 32765898 10.1186/s40900-020-00220-7 PMC7396939

[33] M. Larkin , Z. V. R. Boden , and E. Newton , “On the Brink of Genuinely Collaborative Care,” Qualitative Health Research 25, no. 11 (2015): 1463–1476. 25829467 10.1177/1049732315576494

[34] T. Dimopoulos‐Bick , P. Dawda , R. Verma , and V. Palmer , “Experience‐Based Co‐Design: Tackling Common Challenges,” Journal of Health Design 3, no. 1 (2018): 86–93.

[35] T. L. Dimopoulos‐Bick , C. O'Connor , J. Montgomery , et al., “‘Anyone Can Co‐Design?’: A Case Study Synthesis of Six Experience‐Based Co‐Design (EBCD) Projects for Healthcare Systems Improvement in New South Wales, Australia,” Patient Experience Journal 6, no. 2 (2019): 93–104.

[36] N. Y. Sheikhan , L. D. Hawke , K. Cleverley , et al., “‘It Reshaped How I Will Do Research’: A Qualitative Exploration of Team Members' Experiences With Youth and Family Engagement in a Randomized Controlled Trial,” Health Expectations 24, no. 2 (2021): 589–600. 33587827 10.1111/hex.13206 PMC8077141

[37] S. Bowen , K. McSeveny , E. Lockley , D. Wolstenholme , M. Cobb , and A. Dearden , “How Was It for You? Experiences of Participatory Design in the UK Health Service,” CoDesign 9, no. 4 (2013): 230–246.

[38] Z. Belrhiti , S. Van Belle , and B. Criel , “How Medical Dominance and Interprofessional Conflicts Undermine Patient‐Centred Care in Hospitals: Historical Analysis and Multiple Embedded Case Study in Morocco,” BMJ Global Health 6, no. 7 (2021): e006140. 10.1136/bmjgh-2021-006140 PMC8280911 34261759

[39] S. A. Nancarrow and A. M. Borthwick , “Dynamic Professional Boundaries in the Healthcare Workforce,” Sociology of Health & Illness 27, no. 7 (2005): 897–919. 16313522 10.1111/j.1467-9566.2005.00463.x

[40] G. A. Fine , Tiny Publics: A Theory of Group Action and Culture (Russell Sage Foundation, 2012).

[41] P. Gregory , D. E. Strode , R. AlQaisi , H. Sharp , and L. Barroca , eds., “Onboarding: How Newcomers Integrate Into an Agile Project Team,” in Agile Processes in Software Engineering and Extreme Programming XP 2020 Lecture Notes in Business Information Processing, ed. V. Stray , R. Hoda , M. Paasivaara , and P. Kruchten (Springer, 2020), 383.

[42] T. Green , A. Bonner , L. Teleni , et al., “Use and Reporting of Experience‐Based Codesign Studies in the Healthcare Setting: A Systematic Review,” BMJ Quality & Safety 29 (2020): 64–76. 10.1136/bmjqs-2019-009570 31548278

[43] C. Grindell , E. Coates , L. Croot , and A. O'Cathain , “The Use of Co‐Production, Co‐Design and Co‐Creation to Mobilise Knowledge in the Management of Health Conditions: A Systematic Review,” BMC Health Services Research 22, no. 1 (2022): 877. 35799251 10.1186/s12913-022-08079-y PMC9264579

[44] E. Dent , E. Hoon , A. Kitson , et al., “Translating a Health Service Intervention Into a Rural Setting: Lessons Learned,” BMC Health Services Research 16, no. 62 (2016): 62. 26888017 10.1186/s12913-016-1302-0 PMC4758176

[45] M. Pedler , ed., Action Learning in Practice. ProQuest Ebook Central (Taylor & Francis Group, 2012).

[46] O. Zuber‐Skerritt , “The Concept of Action Learning,” Learning Organization 9, no. 3 (2002): 114–124.

[47] M. Q. Patton , Developmental Evaluation: Applying Complexity Concepts to Enhance Innovation and Use (Guilford Press, 2010).

[48] S. Peters , L. Guccione , J. Francis , et al., “Evaluation of Research Co‐Design in Health: A Systematic Overview of Reviews and Development of a Framework,” Implementation Science 19, no. 1 (2024): 63. 39261956 10.1186/s13012-024-01394-4 PMC11391618

